# The contribution of polymer additives to microplastic toxicity: A long-term study with *Oncorhynchus mykiss* exposed to polystyrene microparticles with different hexabromocyclododecane content

**DOI:** 10.17221/95/2025-VETMED

**Published:** 2026-07-29

**Authors:** Nikola Hodkovicova, Jana Blahova, Kamil Stastny, Michaela Charvatova, Ales Franc, Frantisek Tichy, Lucie Sedlackova, Jan Mares, Martin Faldyna, Zdenka Svobodova

**Affiliations:** ^1^Veterinary Research Institute, Brno, Czech Republic; ^2^Faculty of Veterinary Hygiene and Ecology, University of Veterinary Sciences, Brno, Czech Republic; ^3^Faculty of Pharmacy, Masaryk University, Brno, Czech Republic; ^4^Faculty of Veterinary Medicine, University of Veterinary Sciences Brno, Brno, Czech Republic; ^5^Faculty of AgriSciences, Mendel University in Brno, Brno, Czech Republic

**Keywords:** diet, fish, plastic, pollution, rainbow trout, toxicology

## Abstract

Abstract: Although microplastic toxicity in fish has been extensively studied, the role of polymer additives remains insufficiently understood. This study investigated the combined effects of polystyrene (PS) microplastics and the brominated flame retardant hexabromocyclododecane (HBCD) on the health of rainbow trout (*Oncorhynchus mykiss*) following six weeks of dietary exposure. Fish were exposed to PS particles with varying HBCD content (0.0 mg/g – PS-HBCD_free_, 0.2 mg/g – PS-HBCD_low_, 1.0 mg/g – PS-HBCD_high_) or to HBCD alone (HBCD_only_). The combination of PS and HBCD induced the most pronounced biological responses at molecular, biochemical and histological levels. In the liver, oxidative stress and upregulation of pro-inflammatory cytokines (*il8*, *il2*), together with increased catalase expression, were observed in PS- and/or PS-HBCD-exposed groups, indicating an imbalance in antioxidant defence. Histopathology confirmed liver dystrophy and renal lesions, while elevated vitellogenin expression suggested endocrine disruption. Although HBCD accumulation was confirmed only in the HBCD_only_ group, the polymer-bound form caused comparable physiological alterations, supporting its contribution to combined toxicity. Overall, the study demonstrates that PS microparticles can act as vectors enhancing the bioactivity of embedded additives such as HBCD, resulting in complex multi-organ effects and emphasising the importance of assessing additive–polymer interactions when evaluating the environmental risks of microplastics.

Plastics are synthetic or polysynthetic organic compounds commonly used across industries. Among them, polystyrene (PS) is mainly employed in insulation, food packaging, and electronic equipment (Plastics Europe 2024). The growing production, extensive use, and inadequate waste management of plastics contribute to environmental pollution. Increasing attention has been paid to microplastics – plastic fragments, particles, fibres, or films smaller than 5 mm (Hodkovicova et al. 2022). Fish are key targets for aquatic contamination, as they may ingest microplastics directly or through prey (Jangid et al. 2025).

Microplastic toxicity depends on multiple factors, including concentration, exposure duration, particle size, shape, and polymer type, as well as environmental conditions such as contaminant background, food availability, and the species or developmental stage of the organism (Hodkovicova et al. 2022; Pal et al. 2025). The particle size range used in toxicity studies is a critical experimental consideration. Larger microplastics (<500 µm) represent a substantial fraction of environmentally detected microplastics in freshwater systems and are commonly found in fish gastrointestinal tracts (Lin et al. 2023). Although the physical properties of microplastics are well studied, their chemical composition and additives remain largely overlooked, despite their potential to affect toxicity.

Among the published studies investigating microplastic toxicity in fish, we are aware of only a few (Garrido Gamarro and Costanzo 2022; Bohackova and Cajthaml 2024; Liu et al. 2024) that at least partly consider the presence of additives in plastics as a factor that can potentially contribute to their toxicity. Meanwhile, the global market involves kilotonnes of various plastic additives; they are inherently present in plastic products, and are used intentionally during the manufacturing process to provide the required technological characteristics of final products (Marturano et al. 2017; Wiesinger et al. 2021).

The amounts of additives added to plastic products vary and may reach several tens of percent and, because they are not always chemically bound to polymers, they can be released into the environment (Bridson et al. 2021; Tanaka et al. 2023). This process is mainly influenced by weathering conditions such as water temperature and pH, UV light, or oxygen concentration (Andrade et al. 2021; Hodkovicova et al. 2022).

Brominated flame retardants (BFRs) have been utilised in large quantities in construction applications, electronic equipment, and furniture to help suppress ignition or slow down the process of combustion (BSEF 2021). Among them, hexabromocyclododecane (HBCD), a non-aromatic BFR primarily used in the production of polystyrene plastics (Lopes Marques and Cairrao 2023), is one of the most widely employed. Some of the most widely employed BFRs, such as HBCD, are listed in Annex A of the Stockholm Convention on Persistent Organic Pollutants, which are heavily restricted or phased out in participating countries approving the deal. Therefore, BFRs have recently been replaced (Tao et al. 2023), and much lower quantities of BFRs have been used compared to the past. Despite this, the former extensive usage of BFRs, coupled with their environmental persistence and bioaccumulation potential, can still represent a significant threat to aquatic organisms (Turner 2022).

This study aims to evaluate the toxic effects of PS microplastics with controlled HBCD addition on a model species of freshwater fish, rainbow trout (*Oncorhynchus mykiss*) after long-term dietary exposure. We wanted to determine whether HBCD contained in PS microparticles administered to fish is responsible for the observed toxic effects. For this purpose, fish in our experiments were exposed to three different kinds of PS microparticles containing either no HBCD (PS-HBCD_free_), technologically relevant HBCD concentration (PS-HBCD_high;_ HBCD = 1.0 mg/g) or a concentration five times lower (PS-HBCD_low;_ HBCD = 0.2 mg/g) considered as environmentally relevant. The technologically relevant concentration was selected as a typical of commercial flame-retarded polystyrene products (Mao et al. 2021) while the environmentally relevant concentration representing the lower range of HBCD concentrations detected in environmental microplastics from aquatic systems (Deng et al. 2023). Various indices of the health state of fish were monitored as the hypothesis of this study was that mainly haematological and biochemical parameters, oxidative stress indices, immune profile and endocrine system will be altered.

## Material and methods

### Preparation of PS microparticles containing HBCD

Commercially available non-additivated PS granulate (GP 585A; Synthos, Oświęcim, Poland) and HBCD >95%; mixture of α-, β-, and γ-isomers; CAS 3194-55-6; Merck, Prague, Czech Republic) were used to prepare PS materials with predefined HBCD content at 220 °C. Materials containing either 0 mg/g HBCD (PS-HBCD_free_) or 1.0 mg/g HBCD (PS-HBCD_high_) were prepared using a semi-operational single-screw planetary extruder (Platex, Takimsan, Türkiye) without material retention in the chamber. The extruded materials were crushed using a laboratory crusher (Moretto S.p.A., Massanzago, Italy) and further processed by cryogenic grinding under liquid nitrogen in a pulverising mill. A sieve analysis (Retsch AS 200, Katowice, Poland) was performed to obtain a homogeneous fraction of microparticles sized 200–300 µm. PS-HBCD_low_ microparticles (0.2 mg/g HBCD) were subsequently prepared by mass-mixing appropriate proportions of PS-HBCD_free_ and PS-HBCD_high_ microparticles.

### Characterisation of PS microparticles

The PS-HBCD_free_ microparticles, as well as those containing HBCD (PS-HBCD_low_; PS-HBCD_high_), were characterised. The surface morphology of the particles was first examined under an optical microscope (Leica DVM2500 Digital Camera; Leica Microsystems, Wetzlar, Germany) at 100× and 300× magnification, followed by scanning electron microscopy (SEM) (PhenomPro; Phenom-World B.V., Eindhoven, The Netherlands), operated at an accelerating voltage of 5 kV and magnifications of 150×, 500×, and 1 000×.

HBCD quantification in the particles was performed by high-performance liquid chromatography (HPLC) with UV detection. For standard preparation, an aliquot of the HBCD standard (CAS: 3194-55-6; Merck, Prague, Czech Republic; m = 5.0 mg) was dissolved in 2 ml dichloromethane (≥99.5% stabilised, AnalaR NORMAPUR^®^ analytical reagent; VWR, Radnor, USA). After complete dissolution, 1 ml of the solution was precipitated by adding 2 ml acetonitrile. The mixture was filtered through a 0.45 µm PTFE syringe filter (VWR, USA) and diluted in acetonitrile to prepare calibration standards. The limits of detection (LOD) and quantification (LOQ) were determined from the standard deviation (SD) of the response and the slope of the calibration curve as follows: LOD = (3.3 × SD)/slope; LOQ = (10 × SD)/slope. The calculated limits were LOD = 0.6 mg/l and LOQ = 2.2 mg/l. For sample preparation, 500 mg of microparticles (non-spiked, 0.2 mg/g, and 1 mg/g HBCD-spiked) were dissolved in 3 ml dichloromethane (≥99.5%, stabilised; AnalaR NORMAPUR^®^ analytical reagent; VWR, Radnor, USA). After dissolution of the PS matrix, 0.5 ml of the solution was precipitated by adding 3 ml acetonitrile. Prior to HPLC injection, extracts were filtered through a 0.45 µm PTFE syringe filter (VWR, Radnor, USA).

HPLC analyses were conducted using a Dionex UltiMate 3000 Series system (Thermo Fisher Scientific, Germering, Germany). Chromatographic separation was achieved on a Kinetex 5 µm C18 100 Å column (150 × 4.6 mm; Phenomenex, Torrance, USA) maintained at 35 °C. The mobile phase consisted of acetonitrile and water (80 : 20, v/v) at a flow rate of 1.00 ml/min. The total analysis time was 9 min, with a sample injection volume of 10 µl and an autosampler temperature of 7 °C. HBCD eluted at a retention time of 4.0 min and was detected by UV at 200 nm and 220 nm, with quantification performed at 220 nm. Data acquisition and processing were carried out using Chromeleon v7.2 software (Thermo Fisher Scientific, Germering, Germany). Each sample was analysed in triplicate.

### Preparation and evaluation of feed pellets

Experimental feeds (3.2 kg batches) were prepared using the liquid-addition powder coating technique (Hollerova et al. 2023). Commercial fish feed pellets (Efico E921, 3 mm; Biomar, Brande, Denmark) served as cores and were coated with PS microparticles containing different HBCD levels (PS-HBCD_free_, PS-HBCD_low_, and PS-HBCD_high_), or with coating blends consisting of Starch 1500^®^ (Colorcon, Harleysville, USA) and Aerosil^®^ 200 (Evonik Industries, Essen, Germany), with or without added HBCD. Detailed feed compositions are provided in the Electronic Supplementary Material (ESM) Table S1.

Powders were blended in a high-speed mixer (Stephan UMC5, Hameln, Germany) at 1 500 × *g* for 1 min and applied onto pellets in a coating pan (DKS; Erweka GmbH, Heusenstamm, Germany) with alternating additions of purified water and coating material to enhance adhesion of hydrophobic particles (Garajova et al. 2020). Coated pellets were dried at 50 °C for 4 h in a hot-air oven (Memmert UND 500, Schwabach, Germany). Physical properties were evaluated according to the European Pharmacopoeia for friability, density, and uniformity (Hollerova et al. 2023). Additional analyses included pycnometric density (ATC helium pycnometer; Porotec GmbH, Hofheim am Taunus, Germany), electrostatic charge (JCI 150 Faraday cage and JCI 178 charge measuring unit; Chilworth Technology Ltd., Southampton, UK), moisture content (HX204, Mettler Toledo, Greifensee, Switzerland), and optical (Nikon SZM 1500, Tokyo, Japan) as well as electron microscopy (MIRA 3, Tescan Orsay Holding, Brno, Czech Republic).

### Experimental design

The toxicity study was conducted in compliance with Czech national legislation [Act No. 246/1992 Coll. (Czech Republic 1992); Decree No. 419/2012 Coll. (Czech Republic 2012)] implementing Directive 2010/63/EU of the European Parliament and Council on the protection of animals used for scientific purposes. The procedure followed the modified OECD Test Guideline 215*** **–* Fish, Juvenile Growth Test (OECD 2000). The experimental project was approved by the Ministry of Education, Youth and Sports of the Czech Republic (Decision MSMT-24007/2021-3).

Juvenile *O. mykiss* were obtained from a local hatchery (Hynčice, Czech Republic). A total of 144 fish were randomly distributed into 12 glass tanks (200 l each) connected to a recirculating water system (12 fish per tank; initial mean body weight 97.3 ± 0.2 g). During a 2-week acclimatisation period, fish were fed a commercial diet (Efico E921; 42–45% protein, 24–27% lipid, 13.5–19.5% carbohydrate, 0.8–2.3% fibre, 4.5–6.5% ash, 0.9% phosphorus) three times daily at 1.5% body weight. Water was continuously aerated and maintained at 16.3 ± 0.4 °C, pH 7.0 ± 0.1, and oxygen >90% (9.6 ± 0.3 mg/l). Nitrites, ammonia, and chlorides averaged 0.1 ± 0.1, 0.2 ± 0.1, and 143.5 ± 21.6 mg/l, respectively. The photoperiod was 12 h light/12 h dark, and fish health was monitored three times daily.

After acclimatisation, fish were re-weighed (mean 106.6 ± 2.7 g) to adjust feed rations and assigned to two control and four experimental groups (each in duplicate). Controls received diets without PS or HBCD. Experimental groups were fed as follows: 1) PS microparticles without HBCD (hereinafter referred to as PS-HBCD_free_); 2) PS microparticles with 0.2 mg HBCD/g (hereinafter referred to as PS- HBCD_low_); 3) PS microparticles with 1.0 mg HBCD/g (hereinafter referred to as PS-HBCD_high_); and 4) feed containing only HBCD (1.0 mg/g; HBCD_only_).

Based on previous findings (Hodkovicova et al. 2021), the concentration of PS microparticles in feed was 0.025%. Fish were fed three times daily at 1.5% body weight, and feed doses were recalculated every two weeks. After 6 weeks of exposure, all fish were anaesthetised, blood samples were collected, and individuals were euthanised by a blow to the head followed by spinal transection. Morphometric indices were recorded, and tissues were collected for subsequent analyses.

### Analysis of HBCD content in diet and liver

Diet samples (*n* = 8 per group) were tested prior to exposure to verify HBCD concentrations in each treatment group, while liver samples (*n* = 8 per group) were analysed post-exposure to assess HBCD bioaccumulation. Homogenised samples (0.5 g) were spiked with 50 µl of HBCD standard (1 µg/ml in methanol), vortexed, and extracted with 2 ml acetonitrile containing 1% formic acid. After 20 min of extraction (MSV-3500, Grant Bio) and centrifugation (10 min, 4 °C, 13 500 × *g*), supernatants were frozen (–20 °C, 20 min) and evaporated to dryness under nitrogen (Termovap TV 10; ECOM, Chrášťany, Czech Republic) at 40 °C. Residues were reconstituted in 200 µl methanol/water (70 : 30), centrifuged (10 min, 22 °C, 14 500 × *g*), and transferred to glass vials. Calibration samples (0.1–100 ng/g) were prepared analogously from commercial feed pellets.

Quantification was performed using liquid chromatography–mass spectrometry (LC-MS/MS) on an LC Vanquish Horizon system coupled with a Q Exactive high-resolution mass spectrometer (Thermo Fisher Scientific, San Jose, USA) equipped with an H-ESI source. Separation was achieved on a Kinetex^®^ 1.7 µm HILIC 100 × 2.1 mm column (Phenomenex, Torrance, USA) at a flow rate of 250 µl/min. The mobile phases were (A) 95% H_2_O/5% methanol/0.1% formic acid and (B) 95% methanol/5% H_2_O/0.1% formic acid, using a 15-min gradient from 70% B to 100% B and back. Injection volume was 5 µl, and each sample was analysed in triplicate.

The mass spectrometer operated in negative ion mode (capillary 400 °C; spray voltage 4.5 kV; resolution 70 000; NCE 30). Calibration was performed every two days using multilevel standards, with procedural and solvent blanks included for every eight samples. HBCD was confirmed by monitoring the precursor ion [M−H]^–^ (m/z 636.642 8) and two product ions (m/z 78.918 8 and 80.916 7), and quantified using the most abundant ion (m/z 78.918 8). The limits of detection and quantification were 1.5 ng/g and 4.0 ng/g, respectively. The analytical HBCD standard (CAS 3194-55-6), water, acetonitrile, and methanol were all purchased from Merck (Czech Republic) at ≥99.9% LC-MS/MS purity.

### Morphometric analysis

Following spinal transection, each of the 144 fish was immediately measured for total and standard body length (cm), body weight, and liver weight (g) using a digital balance with a precision of 0.01 g. These data were used to calculate Fulton’s condition factor (FCF) as FCF = [body weight (g)/standard length (cm)^3^] × 100 and the hepatosomatic index (HSI) as HSI = [liver weight (g)/body weight (g)] × 100.

### Haematological and biochemical analysis

For haematological and biochemical analyses, blood was collected from the caudal vein of all fish and heparinised with sodium heparin (50 IU/ml). Haematological parameters (*n = *10 per group) included red blood cell count (RBC), haemoglobin (Hb), packed cell volume (PCV), white blood cell count (WBC), and differential leucocyte counts (lymphocytes, myelocytes, metamyelocytes, band and segmented neutrophils, and monocytes). The neutrophil-to-lymphocyte ratio (N/L) was calculated as a stress indicator. Erythrocyte indices were determined as mean corpuscular haemoglobin (MCH = Hb/RBC), mean corpuscular volume (MCV = PCV/RBC × 1 000), and mean corpuscular haemoglobin concentration (MCHC = Hb/PCV × 1 000), following Svobodova et al. (2012).

Plasma was separated by centrifugation (800 × *g*, 10 min, 4 °C) and analysed for biochemical parameters (*n* = 12 per group). These included protein/nitrogen metabolism (total protein, albumin, ammonia, creatinine), carbohydrate metabolism (glucose, lactate), lipid metabolism (cholesterol, triglycerides), enzyme activities (alkaline phosphatase, alanine aminotransferase, aspartate aminotransferase, lactate dehydrogenase), and mineral levels (phosphorus, magnesium, calcium). Analyses were performed using BioVendor PCL (Brno, Czech Republic) kits and a Konelab 20i biochemical analyser (Thermo Fisher Scientific, Prague, Czech Republic) according to the manufacturers’ instructions.

### Analysis of oxidative stress indicators

Tissue samples of gill, liver, and caudal kidney (*n* = 10 per group) were homogenised in phosphate buffer (pH 7.2; 1 : 10 w/v) and divided into two fractions. One was used for lipid peroxidation assessment by the thiobarbituric acid reactive substances (TBARS) method, and the other was centrifuged (11 000 × *g*, 20 min, 4 °C) to obtain supernatants for spectrophotometric analyses of antioxidant enzymes: superoxide dismutase (SOD), catalase (CAT), glutathione peroxidase (GPx), glutathione reductase (GR), and glutathione-*S*-transferase (GST). GR activity was not measured in kidney tissue due to its low activity. Enzyme activities were normalised to protein concentration determined by a bicinchoninic acid assay (Sigma-Aldrich, St. Louis, USA) using bovine serum albumin as a standard. Detailed methodologies are described in Blahova et al. (2013).

### Analysis of gene expression

During post-mortem examination, tissues including the gill, liver, skin, cranial kidney, and cranial section of the intestine (*n = *10 per group) were collected for subsequent quantitative real-time polymerase chain reaction (qRT-PCR) analysis. The analysis procedure followed Hodkovicova et al. (2021) without modification and the subsequent qPCR analysis was performed in triplicate for each sample. Data were analysed based on threshold cycle (Ct) values using the comparative Δ method. Normalised expression of target genes is presented as mean ± standard deviation (SD) relative to the control.

Primer sequences for the analysed genes were adopted from literature sources cited in ESM Table S2. The mRNA expression of the following genes was evaluated: oxidative stress-related genes (catalase – *cat*; superoxide dismutase types 1 and 2; glutathione-*S*-transferase), apoptosis (caspase 3 – *casp3*), xenobiotic metabolism (cytochrome P450 family 1 subfamily A member 3), immune response (interleukin 1β – *il1β*; interleukin 2 – *il2*; interleukin 8 – *il8*; tumour necrosis factor alpha – *tnfα*), and endocrine disruption markers (thyroid hormone receptor alpha; vitellogenin – *vtg*). Gene expression was normalised using 60S ribosomal protein as the reference gene, selected with NormFinder software v0.953 (MOMA, Denmark).

### Histopathological analysis

For histological examination, tissues including gill (upper arch limb), liver, spleen, cranial and caudal kidney, gonads, and skin (above the ventral fin) were collected from each specimen (*n* = 6 per group). Samples were fixed in 10% neutral buffered formalin, dehydrated in isopropanol, embedded in paraffin, and sectioned at 4 µm using a microtome. Sections were stained with haematoxylin–eosin and examined under a light microscope at 100–400× magnification for morphological evaluation.

### Statistical analysis

Given that two control groups were included in the experiment to account for potential effects associated with different experimental conditions, Control I and Control II were statistically compared using *t*-test. Since no significant differences were observed between the two control groups, they were merged into a single Control group to provide a more robust reference group and were used as one group in all subsequent statistical analyses.

Statistical analyses were performed in Prism v8 (GraphPad Software, USA). Data normality was assessed using the Shapiro–Wilk test and homogeneity of variances using Bartlett’s test. Normally distributed data were analysed by one-way ANOVA followed by Tukey’s multiple comparison test; non-normal data were evaluated using the Kruskal–Wallis test. Differences were considered significant at *P* < 0.05. Results are presented as mean ± SD, and values marked with different superscript letters (a, b, c) indicate statistically significant differences among groups. From the panel of genes analysed for expression (as described in the Material and Methods), many gene–tissue responses did not show statistically significant differences among groups. These non-significant results are therefore not presented or discussed further.

## RESULTS AND DISCUSSION

Despite regulatory restrictions and declining usage, HBCD remains a pervasive contaminant in aquatic environments due to its historic use, bioaccumulation potential and the fact that HBCD continues to appear in microplastic particles retrieved from fresh and marine waters, where it can act both as an additive still contained in polymer matrices and as a chemical that may desorb into the surrounding water (Pittura et al. 2022; Zhang et al. 2022).

Given this dual role of HBCD as both a leachable chemical and a plastic-bound additive, and of PS MPs as vectors, it was essential in our study to test: (1) microparticles of PS without HBCD, (2) the HBCD chemical alone, and (3) PS particles loaded with HBCD. This design allowed us to distinguish between effects attributable to the polymer matrix, to the additive alone, and to their combination.

Before the beginning of the experimental trial, the characterisation of PS particles with different HBCD contents was conducted using optical (ESM Figure S1) and SEM microscopy (ESM Figure S2). The addition of HBCD caused greater particle disintegration compared with the unmodified PS; however, the dominant particle fraction remained within 200–300 µm, as confirmed by SEM analysis (ESM Figure S2). Extraction efficiency was assessed using the same protocol as for the calibration curve solutions. The extraction yield, determined relative to the pure HBCD standard, reached 83%.

Then, prepared samples were serially diluted for HPLC analysis. After back-calculating dilution factors, the following HBCD concentrations were determined: (1) PS-HBCD_free_ group: HBCD not detected in the PS particles; (2) PS-HBCD_low_ group: 0.023 7 ± 0.000 9 % (w/w), i.e., 0.237 mg/g (w/w) HBCD (labelled as 0.2 mg/g for the purpose of this study); (3) PS-HBCD_high_ group: 0.118 4 ± 0.002 5 % (w/w), i.e., 1.184 mg/g (w/w) HBCD (labelled as 1.0 mg/g for the purpose of this study). Detailed results for thermoplastically prepared particle samples and controls are provided in ESM Table S3. Based on these data, we considered the prepared particles, both PS and those with HBCD content, to be suitable for the study.

Also, the prepared feed pellets were assessed for their suitability for experimental purposes. The

physical parameters were evaluated by SEM microscopy (ESM Figure S3) and determination of key physico-chemical properties (ESM Table S4). Additionally, the HPLC analysis of HBCD content in the feed pellets was performed. Analysis of HBCD content in the diet showed <LOQ (i.e., <4.0 ng/g) levels in the control and PS-HBCD_free_ diets. The HBCD concentration in the PS-HBCD_low_ diet was 5.3× lower than in PS-HBCD_high_, whilst the HBCD_only_ diet contained 11.2× higher levels than the PS- HBCD_high_ group. Based on these extensive analyses of the diet, we considered it to be suitable for the experimental study (Table 1).

**Table 1:** HBCD content in diet and liver tissue of *Oncorhynchus mykiss* exposed for six weeks to different PS microparticles (PS-HBCD_free,_ PS-HBCD_low_, PS-HBCD_high_), or HBCD alone (HBCD_only_)

Group	Diet (ng/g)	Liver (ng/g)
Control	<LOQ	<LOQ
PS-HBCD_free_	<LOQ	<LOQ
PS-HBCD_low_	50.0 ± 13.5	<LOQ
PS-HBCD_high_	264.5 ± 108.7	<LOQ
HBCD_only_	2 958.8 ± 62.2	668.7 ± 240.7

Data are given as mean ± SD (n = 8). LOQ = 4.0 ng/g

HBCD = hexabromocyclododecane; HBCD_only_ = feed containing only HBCD in 1.0 mg/g concentration; LOQ = limit of quantification; PS = polystyrene; PS-HBCD_free_ = polystyrene microparticles without HBCD concentration; PS-HBCD_high_ = polystyrene microparticles with 1.0 mg/g concentration of HBCD; PS-HBCD_low_ = polystyrene microparticles with 0.2 mg/g concentration of HBCD

**Figure 1:**
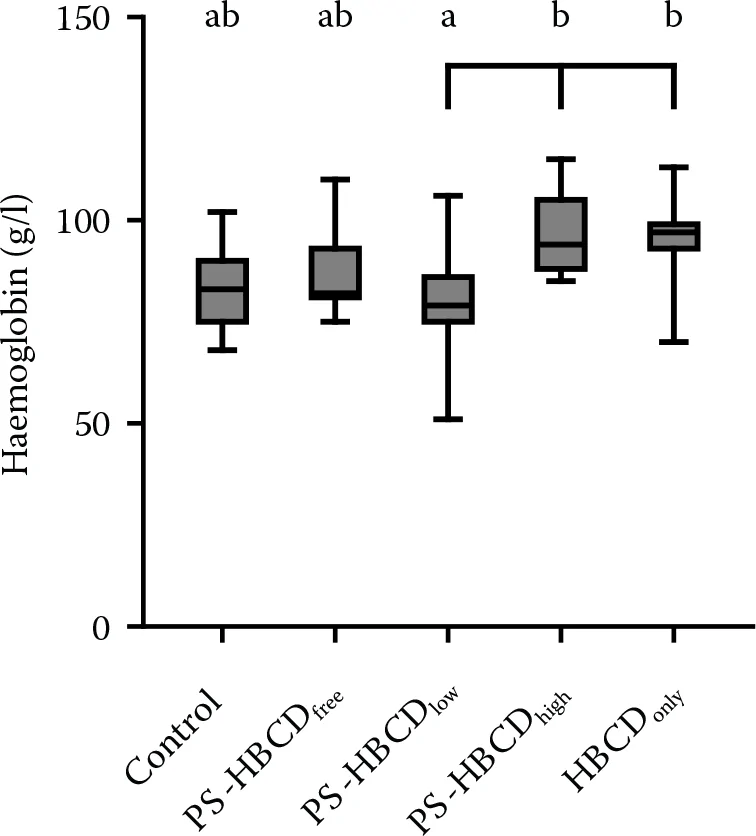
Haemoglobin content in *Oncorhynchus mykiss* blood samples (*n* = 10) after six weeks of oral exposure to PS particles (PS-HBCD_free_), HBCD (HBCD_only_) or their combination – PS-HBCD_low_ and PS-HBCD_high_ ^a,b^Significance was stated as means with different letters of superscript HBCD = hexabromocyclododecane; HBCD_only_ = feed containing only HBCD in 1.0 mg/g concentration; PS = polystyrene; PS-HBCD_free_ = polystyrene microparticles without HBCD concentration; PS-HBCD_high_ = polystyrene microparticles with 1.0 mg/g concentration of HBCD; PS-HBCD_low_ = polystyrene microparticles with 0.2 mg/g concentration of HBCD

During the study, no mortality or growth differences were observed among groups, and morphometric indices remained unchanged. Haematological parameters were largely stable, except for a modest but significant increase in hemoglobin levels (*P* < 0.05) in fish exposed to higher HBCD concentrations (PS-HBCD_high_ and HBCD_only_; Figure 1), suggesting a compensatory response to potential hypoxic or oxidative stress.

**Figure 2:**
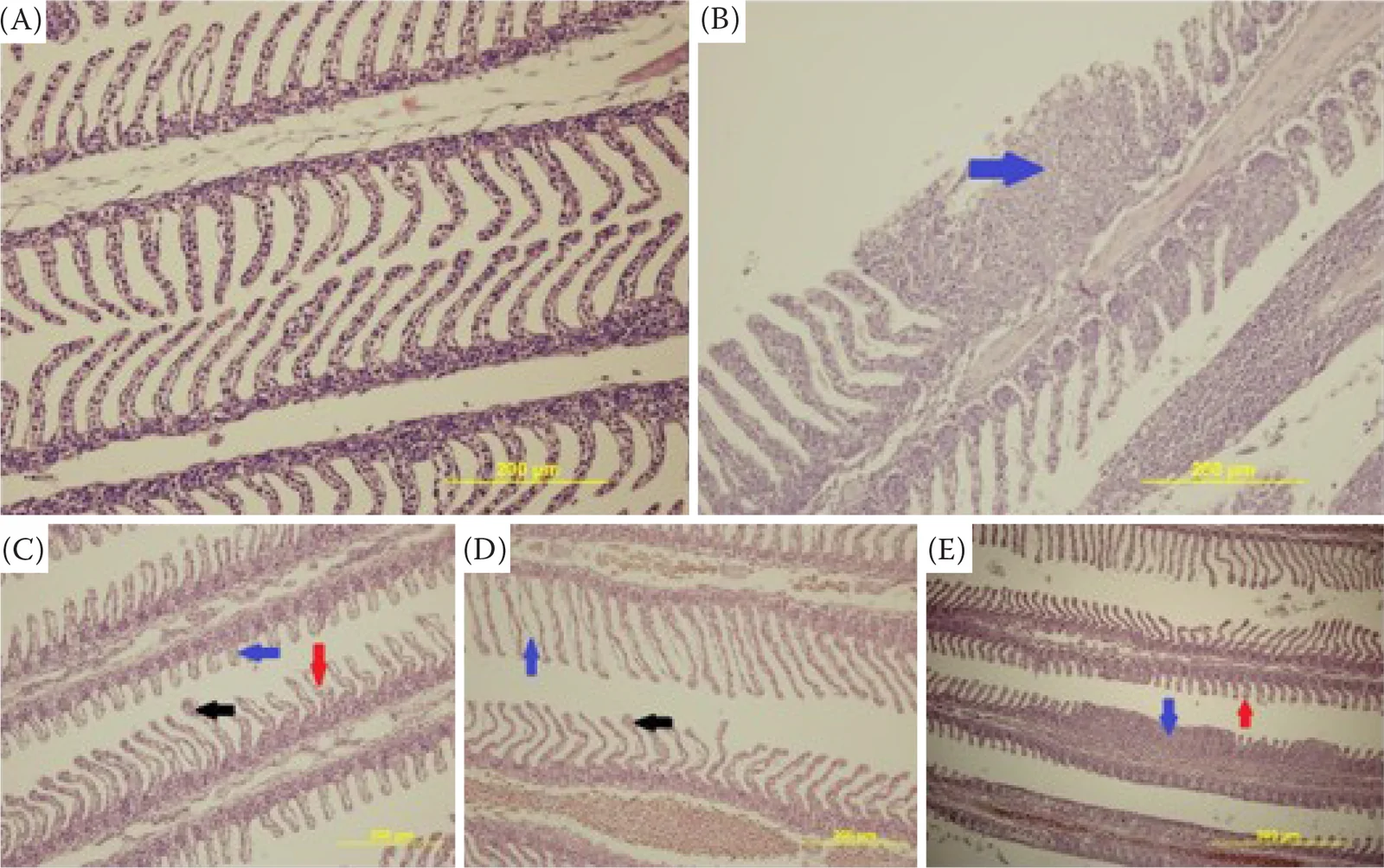
Representative histological pictures of the gills of *Oncorhynchus mykiss* after six weeks of oral exposure to PS particles (PS-HBCD_free_), HBCD (HBCD_only_) or their combination – PS-HBCD_low_ and PS-HBCD_high_. Control group without pathological findings with regular lamellae (A); tissue deformation and presence of inflammatory infiltration (blue arrow) in PS-HBCD_free_ group (B); deformation, shortening (blue arrow), dilatation (red arrow), and inflammatory infiltration (black arrow) in PS-HBCD_low_ group (C); dilatation (blue arrows) and inflammatory infiltration (black arrow) in PS-HBCD_high_ group (D); deformation and shortening (red arrow) and inflammatory infiltration (blue arrows) in HBCD_only_ group (E). Magnification 100–200×, *n = *6/group HBCD = hexabromocyclododecane; HBCD_only_ = feed containing only HBCD in 1.0 mg/g concentration; PS = polystyrene; PS-HBCD_free_ = polystyrene microparticles without HBCD concentration; PS-HBCD_high_ = polystyrene microparticles with 1.0 mg/g concentration of HBCD; PS-HBCD_low_ = polystyrene microparticles with 0.2 mg/g concentration of HBCD

The hypoxic stress could have been caused by damage to gill tissue. Gills exhibited morphological damage in all exposed groups – leaflet dilation, shortening, erosion, and inflammatory infiltration (Figure 2) despite no cytokine upregulation being observed at the molecular level. Prior studies in trout have linked PS exposure to both histological changes and *il1β* upregulation (Hollerova et al. 2023). We hypothesise that the irregular, sharp surfaces of microparticles may physically damage the gill epithelium and thereby overshadow subtler molecular responses, as seen in carp exposed to polyethylene microparticles (Cao et al. 2023).

**Figure 3:**
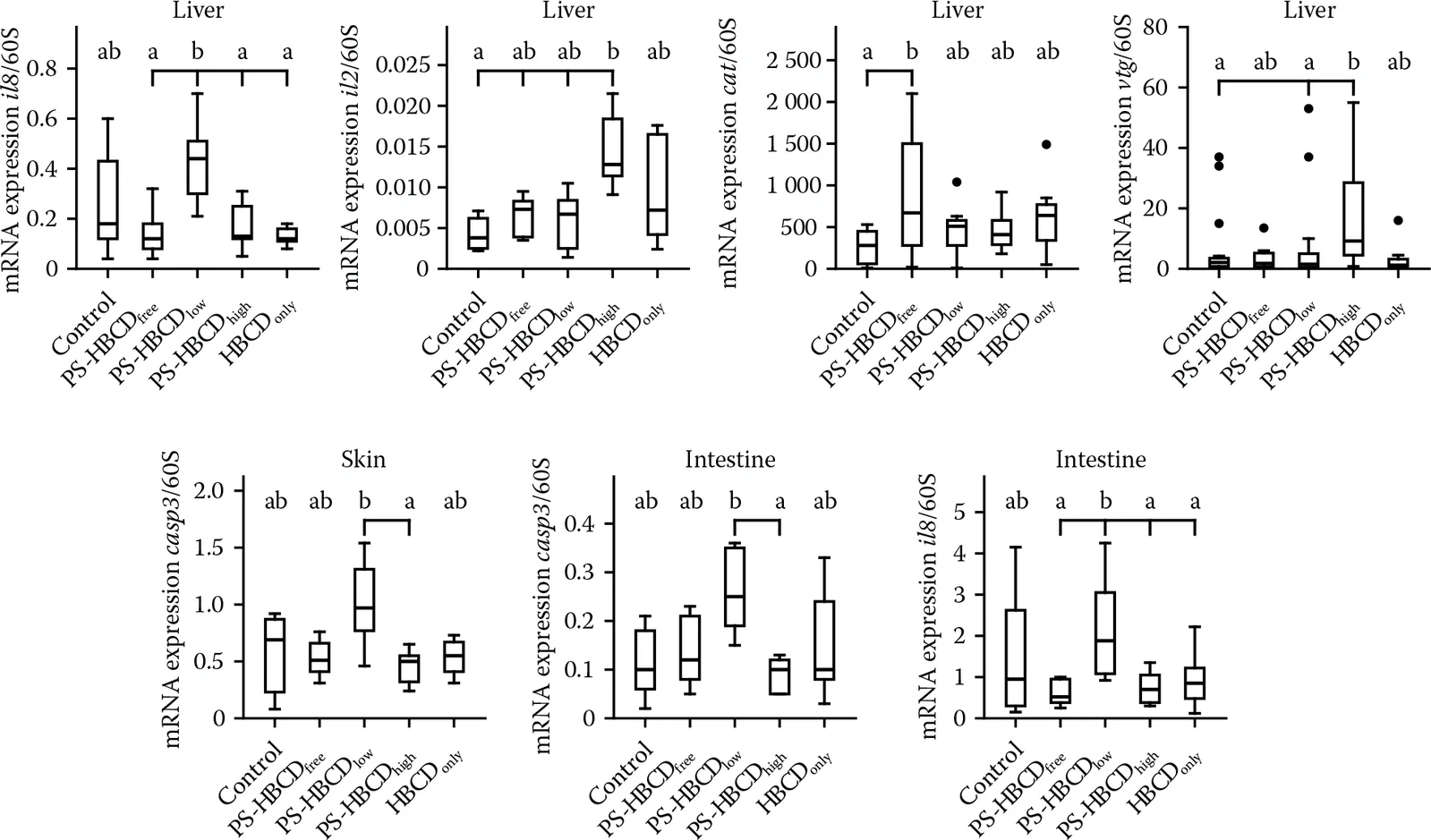
The mRNA expression of genes of interest in the liver, skin and intestine of *Oncorhynchus mykiss* after six weeks of oral exposure to PS particles (PS-HBCD_free_), HBCD (HBCD_only_) or their combination – PS-HBCD_low_ and PS-HBCD_high_ ^a,b^Significance was stated as means with different letters of superscript Data are given as mean ± SD (*n* = 10). Box plot graphs were constructed using median, Q1, and Q3 with whiskers of a maximum 1.5 interquartile range and outliers are denoted as points 60S = 60S ribosomal protein; *casp3* = caspase 3; *cat* = catalase; HBCD = hexabromocyclododecane; HBCD_only_ = feed containing only HBCD in 1.0 mg/g concentration; *il2* = interleukin 2; *il8* = interleukin 8; mRNA = messenger ribonucleic acid; PS = polystyrene; PS-HBCD_free_ = polystyrene microparticles without HBCD concentration; PS-HBCD_high_ = polystyrene microparticles with 1.0 mg/g concentration of HBCD; PS-HBCD_low_ = polystyrene microparticles with 0.2 mg/g concentration of HBCD; Q = quartile; *vtg* = vitellogenin

Most alterations in our study were observed in liver tissue, where molecular signs of inflammation were accompanied by oxidative stress. The mRNA expression of *il8* (in PS-HBCD_low_; *P* < 0.05) and *il2* (in PS-HBCD_high_; *P* < 0.05) was significantly elevated in exposed fish, although the responses varied with HBCD concentration and were tissue-specific (Figure 3). Interleukin-8 functions as a neutrophil chemoattractant, whilst interleukin-2 modulates T-cell proliferation and differentiation (Zou and Secombes 2016). Previous research demonstrated that HBCD can upregulate pro-inflammatory cytokines in human immune cells (Koike et al. 2016; Falconer-Turner et al. 2025), but comparable findings in fish have not been reported to our knowledge. Conversely, hepatic inflammation induced by PS microparticles has been verified in zebrafish and trout (Lu et al. 2016; Hollerova et al. 2023).

In our study, pro-inflammatory gene induction occurred only in PS–HBCD treatments; however, given that polymer-bound HBCD was not detectable in liver tissue, these molecular responses may reflect localised effects or altered particle properties rather than systemic HBCD toxicity. From another perspective, we also detected an increase in *cat* mRNA expression in PS-HBCD_free_ fish (*P* < 0.05), indicating that PS alone may possess toxic potential. Because catalase plays a crucial role in maintaining redox balance, its altered regulation may indicate perturbation of antioxidant defence. Similar redox imbalances have been documented in fish and invertebrates exposed to microplastics and plastic additives (Wan et al. 2019; Roy et al. 2023). Overall, antioxidant patterns in our study appeared inconsistent; the observed molecular response was not accompanied by other oxidative stress markers, suggesting compensatory regulation rather than overt oxidative damage.

The detected concentration of HBCD in the liver was <LOQ in the control, PS-HBCD_free_ group and both groups exposed to a combination of PS and HBCD, i.e. PS-HBCD_low_ and PS-HBCD_high_. Only in the HBCD_only_ group was HBCD accumulation detected in the liver, at an average of ~668.7 ± 240.7 ng/g, confirming that polymer-bound HBCD remains poorly bioavailable (Table 1). This indicates that direct HBCD accumulation cannot explain the observed effects in PS-HBCD groups. The encapsulation within the polymer matrix appears to effectively prevent significant desorption and systemic uptake during our experiment. Wild fish often show liver HBCD in the range of 10–1 000 ng/g wet weight (Haukas et al. 2009). In *Carassius carassius*, HBCD accumulation has been linked to induction of antioxidant enzymes and lipid peroxidation (Dong et al. 2018). Our findings point towards compromised hepatic detoxification following accumulation.

Interestingly, a decrease in plasma ammonia levels was observed in fish exposed to HBCD alone in the absence of PS (Table 2). This finding suggests that HBCD exposure may be associated with altered nitrogen metabolism; however, the mechanism cannot be determined from the present data. Possible explanations include changes in hepatic ammonia detoxification pathways, such as modulation of the urea cycle or glutamine synthesis, or reduced ammonia production due to altered proteolytic activity and amino acid catabolism. These interpretations require targeted biochemical analyses for confirmation. However, such biochemical modulation would suggest that, despite histologically apparent dystrophy, the liver retained or even temporarily upregulated its detoxification capacity as an adaptive response (Smutna et al. 2002; Edwards et al. 2024). Yet histological examination revealed dystrophic alterations in both PS-HBCD_high_ and HBCD_only_ groups (ESM Figure S4), suggesting that morphological damage may precede or occur independently of gene expression changes.

**Table 2:** Biochemical markers in *Oncorhynchus mykiss* blood plasma samples (*n* = 12) after six weeks of dietary exposure to PS particles (PS-HBCD_free_), HBCD (HBCD_only_) or their combination – PS-HBCD_low_ and PS-HBCD_high_

Category	Index (unit)	Control	PS-HBCD_free_	PS-HBCD_low_	PS-HBCD_high_	HBCD_only_
Protein/nitrogen metabolism	TP **(**g/l**)**	50.9 ± 6.8^a^	50.6 ± 3.5^a^	51.5 ± 6.4^a^	51.2 ± 5.7^a^	49.1 ± 5.5^a^
	ALB **(**g/l**)**	19.1 ± 3.3^a^	19.7 ± 1.9^a^	18.7 ± 1.7^a^	20.0 ± 3.0^a^	18.3 ± 3.4^a^
	NH_3_ (µmol/l)	416.2 ± 133.8^ab^	499.7 ± 95.3^ab^	505.6 ± 201.4^ab^	**582.4** ± **180.5^b^**	**387.4** ± **109.8^a^**
	CREA (µmol/l)	31.4 ± 10.1^a^	29.6 ± 7.4^a^	28.1 ± 5.9^a^	29.9 ± 6.3^a^	32.7 ± 8.8^a^
						
Carbohydrate metabolism	GLU (mmol/l)	4.5 ± 1.0^a^	5.2 ± 0.7^a^	5.0 ± 0.7^a^	5.2 ± 0.9^a^	5.3 ± 0.7^a^
	LACT **(**mmol/l**)**	2.9 ± 2.2^a^	3.8 ± 2.2^a^	3.6 ± 2.6^a^	3.9 ± 2.7^a^	2.5 ± 1.3^a^
						
Lipid metabolism	CHOL **(**mmol/l**)**	11.4 ± 2.5^a^	11.4 ± 2.7^a^	11.6 ± 3.0^a^	11.8 ± 1.3^a^	9.8 ± 1.9^a^
	TG **(**nmol/l**)**	3.9 ± 1.9^a^	2.9 ± 0.9^a^	3.7 ± 1.3^a^	3.9 ± 1.3^a^	2.9 ± 0.8^a^
						
Enzyme activities	ALP (µkat/l)	4.8 ± 1.9^a^	5.4 ± 2.7^a^	5.0 ± 1.9^a^	4.8 ± 1.7^a^	3.9 ± 1.3^a^
	ALT (µkat/l)	2.6 ± 1.1^a^	2.7 ± 1.4^a^	1.9 ± 1.0^a^	2.3 ± 1.5^a^	2.6 ± 1.1^a^
	AST (µkat/l)	6.8 ± 2.3^a^	8.8 ± 2.5^a^	7.9 ± 2.4^a^	9.1 ± 2.6^a^	6.6 ± 2.4^a^
	LDH (µkat/l)	17.5 ± 7.2^a^	27.3 ± 15.9^a^	24.1 ± 20.1^a^	29.4 ± 20.4^a^	22.7 ± 12.1^a^
						
Minerals	P **(**mmol/l**)**	3.7 ± 0.2^a^	4.1 ± 0.4^a^	4.1 ± 0.7^a^	4.2 ± 0.7^a^	4.0 ± 0.5^a^
	Mg **(**mmol/l**)**	**1.1** ± **0.2^a^**	1.2 ± 0.1^ab^	1.2 ± 0.1^ab^	**1.3** ± **0.1^b^**	**1.1** ± **0.1^a^**
	Ca **(**mmol/l**)**	2.9 ± 0.2^ab^	2.9 ± 0.3^ab^	2.9 ± 0.2^ab^	**3.1** ± **0.3^b^**	**2.8** ± **0.2^a^**

^a,b^Statistically significant differences (*P* < 0.05) are indicated by different superscript letters and highlighted in bold

ALB = albumin; ALP = alkaline phosphatase; AST = aspartate aminotransferase; ALT = alanine aminotransferase; Ca = calcium; CHOL = cholesterol; CREA = creatinine; GLU = glucose; HBCD = hexabromocyclododecane; HBCD_only_ = feed containing only HBCD in 1.0 mg/g concentration; LACT = lactate; LDH = lactate dehydrogenase; Mg = magnesium; NH_3_ = ammonia; P = phosphorus; PS = polystyrene; PS-HBCD_free_ = polystyrene microparticles without HBCD concentration; PS-HBCD_high_ = polystyrene microparticles with 1.0 mg/g concentration of HBCD; PS-HBCD_low_ = polystyrene microparticles with 0.2 mg/g concentration of HBCD; TG = triglycerides; TP = total protein

It is important to acknowledge a critical limitation of our experiment. Whilst we observed biological responses in fish exposed to PS-HBCD combinations compared to PS alone or HBCD alone, the absence of detectable HBCD accumulation in liver tissue from PS-HBCD groups prevents definitive attribution of effects to the additive itself. The inclusion of HBCD during thermoplastic processing resulted in particles with altered physical characteristics, including increased fragmentation and potentially modified surface properties. These changes in particle morphology and behaviour may independently contribute to toxicity through enhanced epithelial damage or altered cellular uptake kinetics. Future studies employing radiolabelled HBCD, gut tissue analysis, and particle surface characterisation would help resolve the observed effects in greater detail (Hermabessiere et al. 2017; Tanaka et al. 2023).

Beyond the liver, we observed evidence of gut–liver axis and renal disturbances. In other fish models, ingestion of microplastics has been shown to alter microbiome composition and disrupt metabolic homeostasis (Wan et al. 2019; Feng et al. 2021; Liao et al. 2023). Though we did not assess the microbiota, intestinal upregulation of *il8* and *casp3* (both increased in the intestinal tissue of the PS-HBCD_low_ group; *casp3* mRNA expression also increased in skin tissue of the PS-HBCD_low_) may suggest localised inflammation and apoptotic signalling (Figure 3). These molecular changes were not accompanied by severe histopathological alterations in the intestine in our study; however, this could support hypotheses that brominated flame retardants may induce endoplasmic reticulum stress and apoptosis (Bertucci et al. 2020; Mohammed et al. 2023). Given the intestine’s role in mineral absorption and osmoregulation, the observed plasma disturbances in magnesium (elevated in the PS-HBCD_high_ group compared to the control; *P* < 0.05) and calcium (elevated in the PS-HBCD_high_ group compared to the HBCD_only_; *P* < 0.01) may reflect systemic ion dysregulation (Table 2). These findings are consistent with our previous findings in trout exposed to microplastics (Hodkovicova et al. 2021).

**Figure 4:**
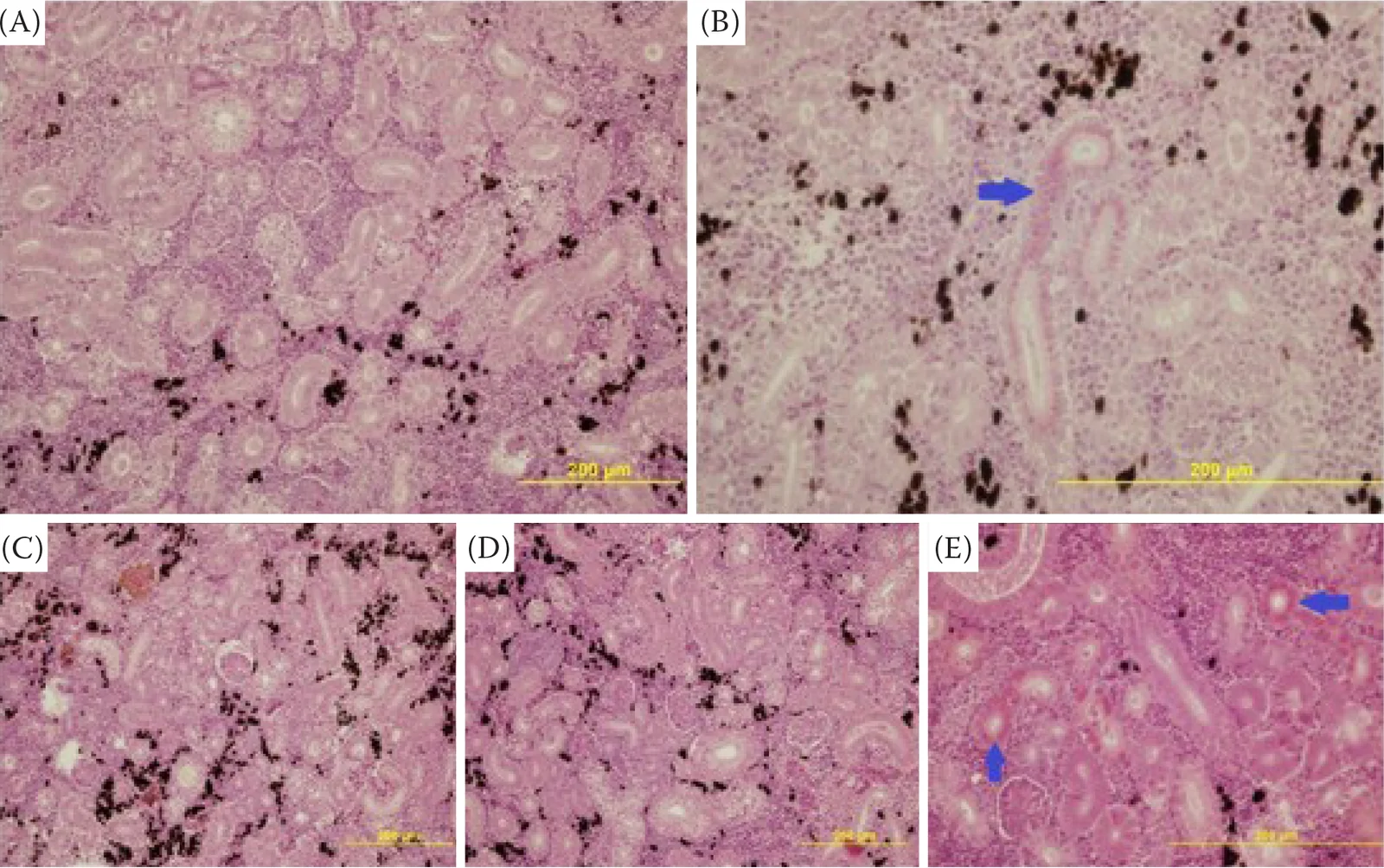
Representative histological pictures of the caudal kidney of *Oncorhynchus mykiss* after six weeks of oral exposure to PS particles (PS-HBCD_free_), HBCD (HBCD_only_) or their combination – PS-HBCD_low_ and PS-HBCD_high_ Mild tissue congestion and local pigment deposits in control (A); areas of hyalinosis of the tubular lining (blue arrows) in the PS-HBCD_free_ group (B); PS-HBCD_low_ (C); and PS-HBCD_high_ group (D); and HBCD_only_ group (E). Magnification 100–200×, *n* = 6/group HBCD = hexabromocyclododecane; HBCD_only_ = feed containing only HBCD in 1.0 mg/g concentration; PS = polystyrene; PS-HBCD_free_ = polystyrene microparticles without HBCD concentration; PS-HBCD_high_ = polystyrene microparticles with 1.0 mg/g concentration of HBCD; PS-HBCD_low_ = polystyrene microparticles with 0.2 mg/g concentration of HBCD

Renal histology further supported this view: tubular hyalinosis was present in PS-HBCD_free_ and HBCD_only_ groups (Figure 4), consistent with homeostatic disruption and degenerative changes previously seen in trout exposed to PS microparticles (Hodkovicova et al. 2021). Thus, kidney lesions likely contributed to electrolyte imbalance. In the cranial kidney, congestion of the organ with small foci of pigment deposits was present in the control group as well as in the PS-HBCD_low_ and PS-HBCD_free_ groups. Pigment deposits were more common in the HBCD_only_ and PS-HBCD_high_ groups (ESM Figure S5).

We also identified potential endocrine-disrupting effects: *vtg* mRNA expression increased in the PS- HBCD_high_ group compared to the control (*P < *0.05) and, moreover, compared to the PS-HBCD_low_ group (*P* < 0.01) (Figure 3). Vitellogenin is a classical biomarker of estrogenic exposure and oviparous disruption (Weiserova et al. 2023). Similar upregulation of *vtg* in fish exposed to microparticles has been documented (Mak et al. 2019; Fernandez-Miguez et al. 2023). Whilst we observed this effect specifically in the PS-HBCD_high_ group, the absence of liver HBCD accumulation in PS-HBCD combinations suggests that if HBCD contributes to this response, it does so through non-systemic mechanisms. The absence of corresponding histopathological changes in gonadal tissue in our study suggests this molecular response may represent an early indicator rather than reproductive toxicity. The observation of an effect caused by HBCD embedded in PS merits further investigation.

Overall, our data provide insight into dietary exposure to PS microplastics with HBCD in freshwater fish. The liver stands as the focal point of combined oxidative, inflammatory, and metabolic stress, whilst the combination of polymer and additive appears to be associated with broader endocrine and renal responses than those observed following single exposures. Whilst combined PS and HBCD treatments elicited broader molecular responses than single exposures, the present study was not designed to formally test interaction terms using a factorial or interaction-focused statistical approach. Importantly, PS-HBCD combinations elicited distinct biological responses compared to PS alone or HBCD alone; however, the absence of detectable liver HBCD accumulation in PS-HBCD groups indicates that these effects cannot be attributed to systemic additive toxicity. Although PS alone induced oxidative reactions and HBCD alone altered hematological and biochemical parameters, only PS-HBCD_high_ induced consistent multi-level responses across molecular, physiological, and histological endpoints.

Notably, responses were often stronger in PS- HBCD_low_ than in PS-HBCD_high_ fish – mirroring inverted dose–response phenomena reported in *Pimephales promelas* explants and HepG2 cell lines (Wang et al. 2016; Bertucci et al. 2020). This may reflect the activation of protective pathways at higher exposures that blunt marker expression, whereas lower exposures fall below adaptive thresholds. Because the PS- HBCD_low_ dose is ecologically relevant, its pronounced response suggests a potential sublethal hazard at environmentally realistic concentrations. We hypothesise that excessive xenobiotic load in PS- HBCD_high_ may suppress defence signalling, leading to flattened molecular responses, an effect observed previously in trout exposed to polyethylene microplastics (Hodkovicova et al. 2021).

In future studies, we propose integrating proteomics, metabolomics, and transcriptomics to disentangle the mechanisms of carrier-mediated toxicity of microplastics with additives. We also suggest investigating long-term, multi-generation impacts to assess cumulative risks.

This study examined the toxicity of polystyrene microplastics with and without hexabromocyclododecane (HBCD) in rainbow trout under controlled laboratory conditions. Under the experimental conditions tested (200–300 µm particles, dietary exposure), PS-HBCD combinations induced multi-organ responses including hepatic oxidative stress, pro-inflammatory cytokine modulation, and endocrine disruption. Notably, HBCD accumulation in liver tissue was detected only when administered as a free chemical, not when bound to PS particles, indicating poor bioavailability of polymer-encapsulated additives. The absence of detectable liver HBCD in PS-HBCD groups prevents definitive attribution of the observed effects to systemic additive toxicity; alternative mechanisms including altered particle properties, localised gut effects, or low-level leaching below detection limits require further investigation. Whilst PS-HBCD_high_ treatment showed the most consistent responses across molecular, biochemical, and histological endpoints, many molecular changes exhibited tissue-specific patterns and modest effect sizes, suggesting adaptive rather than severe toxic responses. These findings demonstrate that plastic-additive interactions can elicit biological responses distinct from either component alone, though the mechanisms remain incompletely resolved. Extrapolation to environmental risk assessment should account for limitations including particle size range, controlled exposure route, lack of demonstrated additive bioaccumulation in combined treatments, and the model species used. Future research integrating gut tissue analysis, particle surface characterisation, and multi-generational studies is needed to resolve mechanistic uncertainties and assess ecological relevance across diverse exposure scenarios.

## Supplementary Material

Supplementary Files
