# Supplementary material for: The contribution of polymer additives to microplastic toxicity: A long-term study with Oncorhynchus mykiss exposed to polystyrene microparticles with different hexabromocyclododecane content
Source: Vet Med (Praha). 2026 Jul 29;71(7):280–94. doi: 10.17221/95/2025-VETMED (PMC13501264; doi:10.17221/95/2025-VETMED)
Supplement: Supplementary file 1 [file VETMED-71-07-125095-s001.pdf]

https://doi.org/10.17221/95/2025-VETMED

The contribution of polymer additives to microplastic toxicity: A long-term study with *Oncorhynchus mykiss* exposed to polystyrene microparticles with different hexabromocyclododecane content

Nikola Hodkovicova[1], Jana Blahova[2], Kamil Stastny[1], Michaela Charvatova[1], Ales Franc[3], Frantisek Tichy[4], Lucie Sedlackova[3], Jan Mares[5], Martin Faldyna[1], Zdenka Svobodova[2]*

[1]*Veterinary Research Institute, Brno, Czech Republic*
[2]*Faculty of Veterinary Hygiene and Ecology, University of Veterinary Sciences, Brno, Czech Republic*
[3]*Faculty of Pharmacy, Masaryk University, Brno, Czech Republic*
[4]*Faculty of Veterinary Medicine, University of Veterinary Sciences Brno, Brno, Czech Republic*
[5]*Faculty of AgriSciences, Mendel University in Brno, Brno, Czech Republic*

**Corresponding author: nikola.hodkovicova@vri.cz*

The authors are fully responsible for both the content and the formal aspects of the electronic supplementary material. No editorial adjustments were made.

Electronic Supplementary Material (ESM)

Table S1. Composition of experimental feeds used throughout the exposure period
Table S2. List of primers used for real-time quantitative polymerase chain reaction
Table S3. Results of HPLC determination of the mass concentration of HBCD in particles
Table S4. Physico-chemical properties of feeds used throughout the adaptation and experiment
Figure S1. Optical microscope images of pure PS particles
Figure S2. Electron microscope images of pure PS particles
Figure S3. SEM images of the feeds used throughout the adaptation and experiment
Figure S4. Representative histological pictures of the liver of *O. mykiss*
Figure S5. Representative histological pictures of the cranial kidney of *O. mykiss*

https://doi.org/10.17221/95/2025-VETMED

Table S1. Composition of experimental feeds used throughout the exposure period

Sample (g)	Control I	Control II	PS-HBCD$_{free}$	PS-HBCD$_{low}$	PS-HBCD$_{high}$	HBCD$_{only}$
Feed pellets	3 200.0	3 104.0	3 136.0	3 136.0	3 136.0	3 103.4
PS-HBCD$_{free}$ particles	–	–	64.0	–	–	–
PS-HBCD$_{low}$ particles	–	–	–	64.0	–	–
PS-HBCD$_{high}$ particles	–	–	–	–	64.0	–
Blend	–	96.0	–	–	–	–
Blend-HBCD	–	–	–	–	–	96.6
Purified water	60.0	160.0	60.0	60.0	60.0	160.0
Total	3 260.0	3 360.0	3 260.0	3 260.0	3 260.0	3 360.0

Commercial feed pellets (EFICO Enviro 921, 3 mm; Biomar, Denmark) were used as cores that were coated by liquid-addition powder coating technology with suitable material (PS microparticles, Blend, Blend-HBCD). The coating material "Blend-HBCD" was prepared by mixing Starch 1500® (Colorcon, USA), Aerosil® 200 (Evonik Industries, Germany) and HBCD, whereas "Blend" contained a mix of Starch 1500® and Aerosil® 200 only. In the end of the coating process, purified water was completely evaporated from all the feed pellets

HBCD = hexabromocyclododecane; HBCD$_{only}$ = feed containing only HBCD in 1.0 mg/g concentration; PS = polystyrene; PS-HBCD$_{free}$ = polystyrene microparticles without HBCD concentration; PS-HBCD$_{high}$ = polystyrene microparticles with 1.0 mg/g concentration of HBCD; PS-HBCD$_{low}$ = polystyrene microparticles with 0.2 mg/g concentration of HBCD

https://doi.org/10.17221/95/2025-VETMED

Table S2. List of primers used for real-time quantitative polymerase chain reaction

Primer	Gene name	Function	Sequence (5'-3')	Reference
60S	60S ribosomal protein	house-keeping gene	F: AGCCACCAGTATGCTAACCAGT<br>R: TGTGATTGCACATTGACAAAAA	Hodkovicova et al. (2021)
cat	catalase	oxidative stress	F: TGATGTCACACAGGTGCGTA<br>R: GTGGGCTCAGTGTTGTTGAG	Meiler et al. (2021)
sod1	superoxide dismutase type 1	oxidative stress	F: TGGTCCTGTGAAGCTGATTG<br>R: TTGTCAGCTCCTGCAGTCAC	Meiler et al. (2021)
sod2	superoxide dismutase type 2	oxidative stress	F: TCCCTGACCTGACCTACGAC<br>R: GGCCTCCTCCATTAAACCTC	Meiler et al. (2021)
gst	glutathione *S*-transferase	oxidative stress	F: TCGCTGACTGGACGAAAGGA<br>R: CGAAGGTCCTCAACGCCATC	Meiler et al. (2021)
casp3	caspase 3	apoptosis	F: AGCTGCTTTACACTGTGTCTCAA<br>R: GCTCAGCATCACACACACAAAG	Foltz et al. (2009)
cyp1a3	cytochrome P450 family 1 subfamily A member 3	xenobiotic metabolism	F: CCCCTTCCGCCATATTGTC<br>R: CGGCCGAAGCACATTCC	Skillman et al. (2006)
il1β	interleukin 1 beta	immune response	F: ACATTGCCAACCTCATCATCG<br>R: TTGAGCAGGTCCTTGTCCTTG	Hodkovicova et al. (2021)
il2	interleukin 2	immune response	F: ATGCAACACCACATCAGCATGAC<br>R: TGTCCGTTGTGCTGTTCTCCTTA	Hodkovicova et al. (2021)
il8	interleukin 8	immune response	F: AGAATGTCAGCCAGCCTTGT<br>R: TCTCAGACTCATCCCCTCAGT	Hodkovicova et al. (2021)
tnfα	tumour necrosis factor alpha	immune response	F: GGGGACAAACTGTGGACTGA<br>R: GAAGTTCTTGCCCTGCTCTG	Hodkovicova et al. (2021)
thrα	thyroid hormone receptor alpha	marker of endocrine disruption	F: TGGGAGCTGATCCGCATG<br>R: CCTTGTCTCCGTCCGAC	Quesada-Garcia et al. (2014)
vtg	vitellogenin	marker of endocrine disruption	F: TGTAGCTTGTGAACCCTGAGA<br>R: TACCCACAACACCCTTGGCATACT	Giraudo et al. (2017)

60S= 60S ribosomal protein; *casp3* = caspase 3; *cat* = catalase; F = forward primer; HBCD = hexabromocyclododecane; $HBCD_{only}$ = feed containing only HBCD in 1.0 mg/g concentration; *il2* = interleukin 2; *il8* = interleukin 8; mRNA = messenger ribonucleic acid; PS = polystyrene; PS-$HBCD_{free}$ = polystyrene microparticles without HBCD concentration; PS-$HBCD_{high}$ = polystyrene microparticles with 1.0 mg/g concentration of HBCD; PS-$HBCD_{low}$ = polystyrene microparticles with 0.2 mg/g concentration of HBCD; Q = quartile; R = reverse primer; *vtg* = vitellogenin

https://doi.org/10.17221/95/2025-VETMED

Table S3. Results of HPLC determination of the mass concentration of HBCD in a sample of PS particles without HBCD addition (PS-$HBCD_{free}$); PS particles with 0.2 mg/g of HBCD (PS-$HBCD_{low}$); PS particles with 1.0 mg/g of HBCD (PS-$HBCD_{high}$)

Sample	Mass concentration of HBCD in PS (% w/w)	Mass concentration of HBCD in PS (mg/g)
PS-$HBCD_{free}$	–	–
PS-$HBCD_{low}$	0.023 7 ± 0.000 9	0.237
PS-$HBCD_{high}$	0.118 4 ± 0.002 5	1.184

HBCD = hexabromocyclododecane; $HBCD_{only}$ = feed containing only HBCD in 1.0 mg/g concentration; PS = polystyrene; PS-$HBCD_{free}$ = polystyrene microparticles without HBCD concentration; PS-$HBCD_{high}$ = polystyrene microparticles with 1.0 mg/g concentration of HBCD; PS-$HBCD_{low}$ = polystyrene microparticles with 0.2 mg/g concentration of HBCD

Table S4. Physico-chemical properties of feeds used throughout the adaptation (commercial feed pellets EFICO Enviro 921, 3 mm; Biomar, Denmark ~ cores) and the exposure period

Parameter (unit)	Cores	Control I	Control II	PS-$HBCD_{free}$	PS-$HBCD_{low}$	PS-$HBCD_{high}$	$HBCD_{only}$
Friability (%)	0.52	0.37	1.34	0.63	0.78	0.54	1.24
Bulk density (g/ml)	0.49 ± 0.01	0.56 ± 0.00	0.51 ± 0.02	0.53 ± 0.01	0.54 ± 0.02	0.54 ± 0.01	0.50 ± 0.00
Tapped density (g/ml)	0.52 ± 0.01	0.58 ± 0.01	0.53 ± 0.00	0.56 ± 0.00	0.57 ± 0.00	0.57 ± 0.00	0.52 ± 0.01
Hausner ratio	1.05	1.04	1.04	1.05	1.06	1.07	1.04
Minimal mass (mg)	21.30	24.93	2.20	23.40	24.40	23.00	25.90
Maximal mass (mg)	23.80	28.70	28.80	26.80	28.90	27.60	28.60
Average mass (mg)	22.61 ± 0.81	24.93 ± 1.74	27.21 ± 1.16	25.13 ± 1.12	26.44 ± 1.36	25.52 ± 1.48	27.07 ± 0.87
RSD of mass (%)	3.58	6.98	4.26	4.46	5.14	5.80	3.21
Pycnometric density (g/ml)	1.22 ± 0.01	1.22 ± 0.00	1.21 ± 0.00	1.21 ± 0.00	1.21 ± 0.00	1.23 ± 0.00	1.21 ± 0.00
Charge (nC)	0.76 ± 0.10	0.56 ± 0.09	–4.13 ± 0.26	–0.10 ± 0.04	–1.24 ± 0.21	–0.47 ± 0.10	–5.77 ± 0.36
Moisture content (%)	1.30 ± 0.04	0.59 ± 0.11	0.89 ± 0.11	0.79 ± 0.10	0.72 ± 0.05	0.66 ± 0.06	1.45 ± 0.11
Perimeter (mm)	14.29	13.55	14.47	15.06	14.81	14.92	14.44
Length (mm)	4.82	4.33	4.69	5.14	5.01	5.11	4.78
Width (mm)	2.37	2.65	2.60	2.40	2.42	2.36	2.51
Maximal projection (mm)	4.41	4.35	4.46	4.48	4.44	4.43	4.42
Minimal projection (mm)	3.34	3.48	3.59	3.62	3.60	3.54	3.49

HBCD = hexabromocyclododecane; $HBCD_{only}$ = feed containing only HBCD in 1.0 mg/g concentration; PS = polystyrene; PS-$HBCD_{free}$ = polystyrene microparticles without HBCD concentration; PS-$HBCD_{high}$ = polystyrene microparticles with 1.0 mg/g concentration of HBCD; PS-$HBCD_{low}$ = polystyrene microparticles with 0.2 mg/g concentration of HBCD

https://doi.org/10.17221/95/2025-VETMED

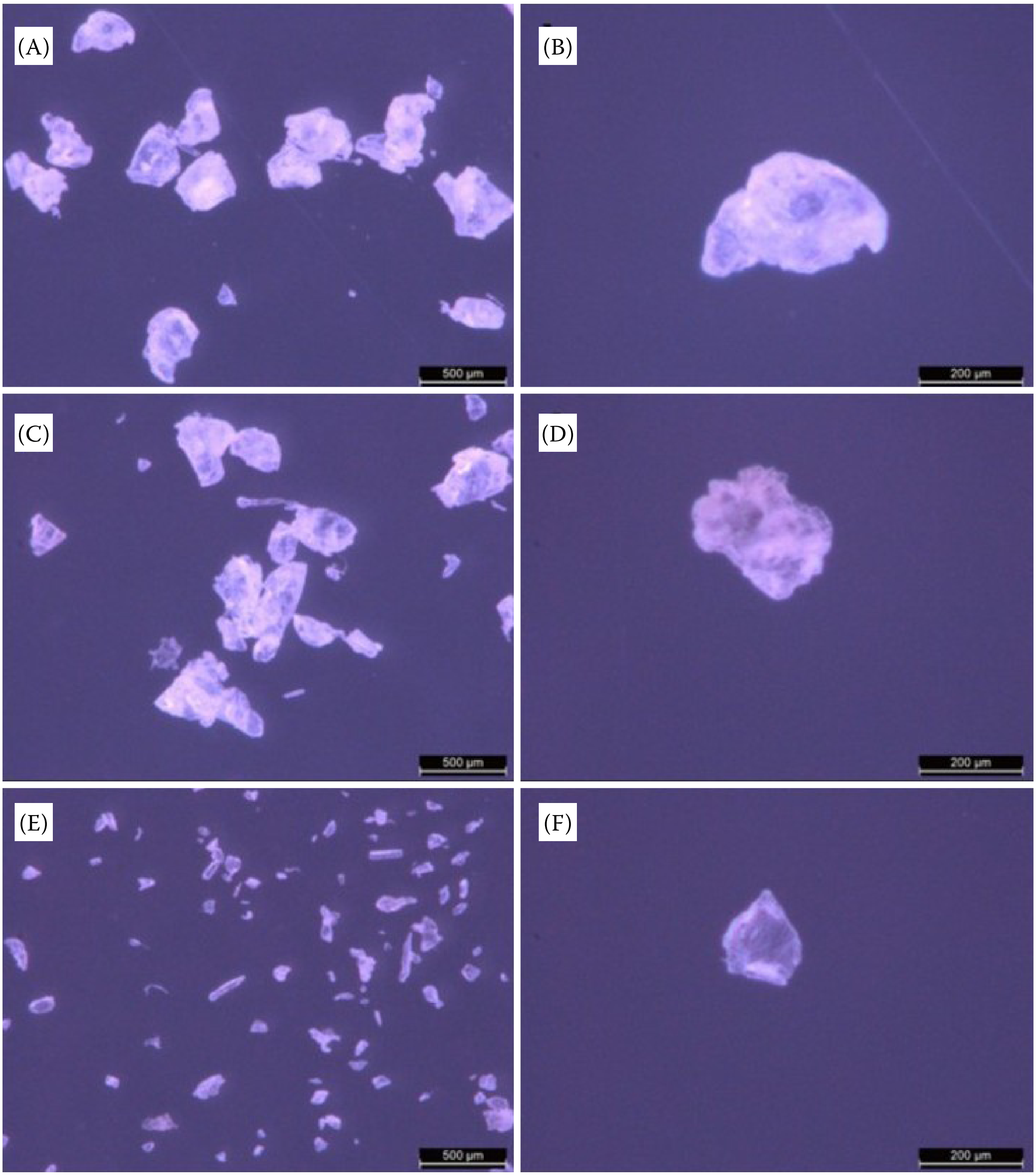

Figure S1. Optical microscope images of pure PS particles without HBCD – PS-$HBCD_{free}$ group (A, B); and images of PS particles containing 0.2 mg/g – PS-$HBCD_{low}$ group (C, D) and 1.0 mg/g HBCD – PS-$HBCD_{high}$ group (E, F). Magnification 100× with a scale of 500 µm (left), and 300× with a scale of 200 µm (right)

HBCD = hexabromocyclododecane; $HBCD_{only}$ = feed containing only HBCD in 1.0 mg/g concentration; PS = polystyrene; PS-$HBCD_{free}$ = polystyrene microparticles without HBCD concentration; PS-$HBCD_{high}$ = polystyrene microparticles with 1.0 mg/g concentration of HBCD; PS-$HBCD_{low}$ = polystyrene microparticles with 0.2 mg/g concentration of HBCD

https://doi.org/10.17221/95/2025-VETMED

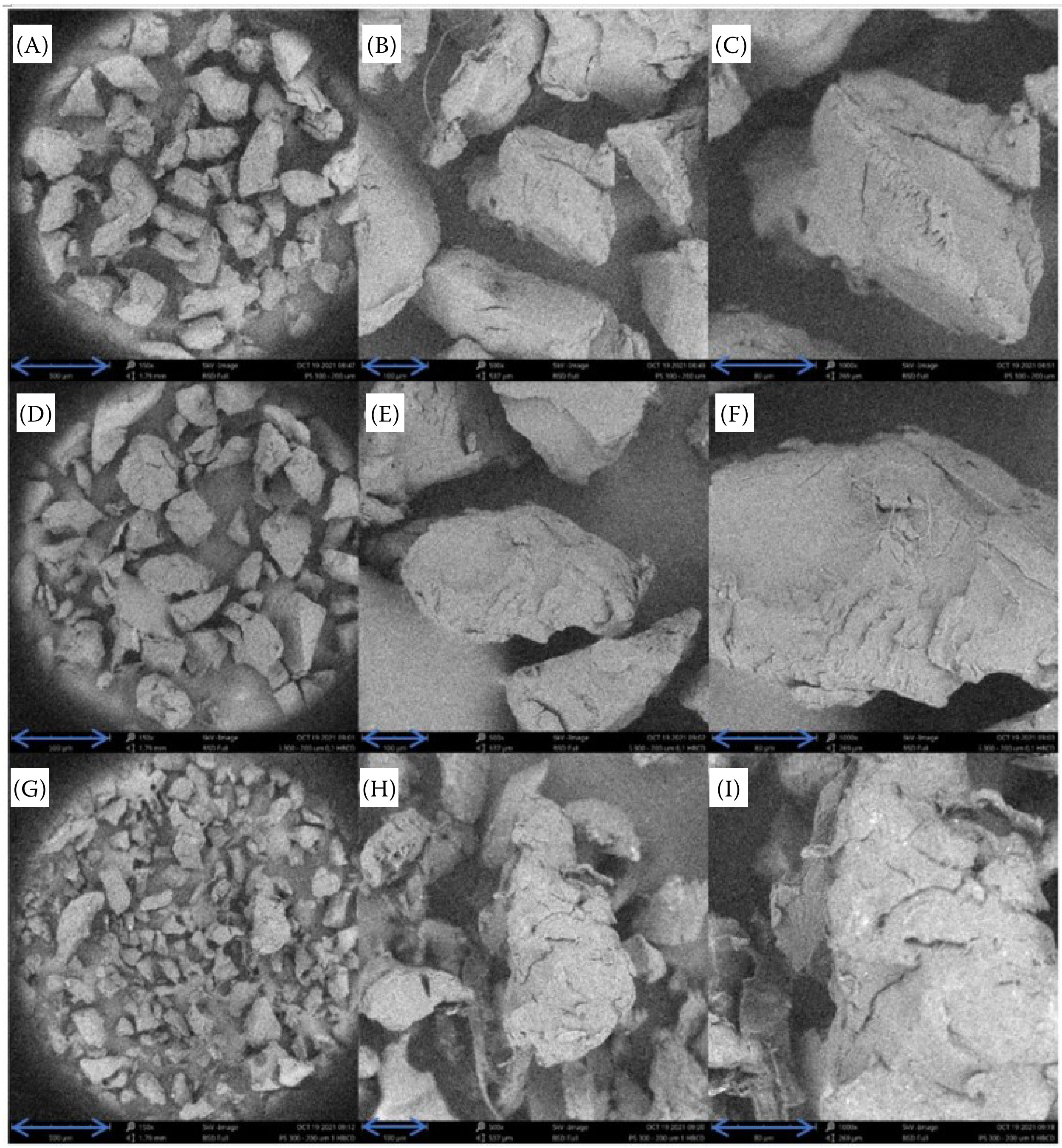

Figure S2. Electron microscope images of pure PS particles without HBCD – PS-$HBCD_{free}$ group (A, B, C); and images of PS particles containing 0.2 mg/g – PS-$HBCD_{low}$ group (D, E, F) and 1.0 mg/g HBCD – PS-$HBCD_{high}$ group (G, H, I). Magnification 150× with a scale of 500 μm (left); magnification 500× with a scale of 100 μm (middle); and magnification 1 000× with a scale of 80 μm (right)

HBCD = hexabromocyclododecane; $HBCD_{only}$ = feed containing only HBCD in 1.0 mg/g concentration; PS = polystyrene; PS-$HBCD_{free}$ = polystyrene microparticles without HBCD concentration; PS-$HBCD_{high}$ = polystyrene microparticles with 1.0 mg/g concentration of HBCD; PS-$HBCD_{low}$ = polystyrene microparticles with 0.2 mg/g concentration of HBCD

https://doi.org/10.17221/95/2025-VETMED

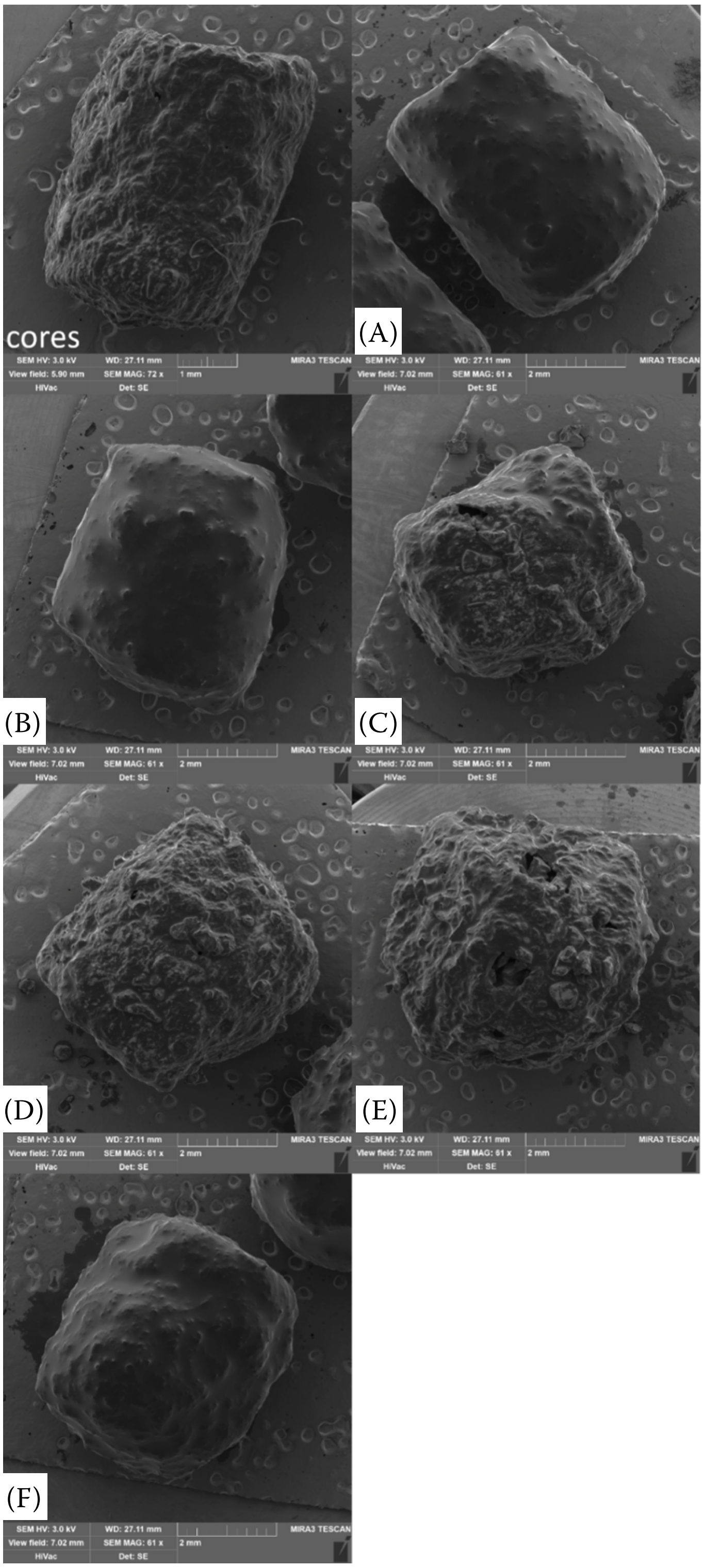

Figure S3. SEM images of the feeds used throughout the adaptation (commercial feed pellets ~ cores) and the exposure period. (A) Control I; (B) Control II; (C) PS-$HBCD_{free}$ (i.e. PS 2 g/kg); (D) PS-$HBCD_{low}$ (i.e. PS 2 g/kg with HBCD 0.2 mg/g); (E) PS-$HBCD_{high}$ (i.e. PS 2 g/kg with HBCD 1.0 mg/g); (F) $HBCD_{only}$ (i.e. HBCD 1.0 mg/g)

HBCD = hexabromocyclododecane; $HBCD_{only}$ = feed containing only HBCD in 1.0 mg/g concentration; PS polystyrene; PS-$HBCD_{free}$ = polystyrene microparticles without HBCD concentration; PS-$HBCD_{high}$ = polystyrene microparticles with 1.0 mg/g concentration of HBCD; PS-$HBCD_{low}$ = polystyrene microparticles with 0.2 mg/g concentration of HBCD

https://doi.org/10.17221/95/2025-VETMED

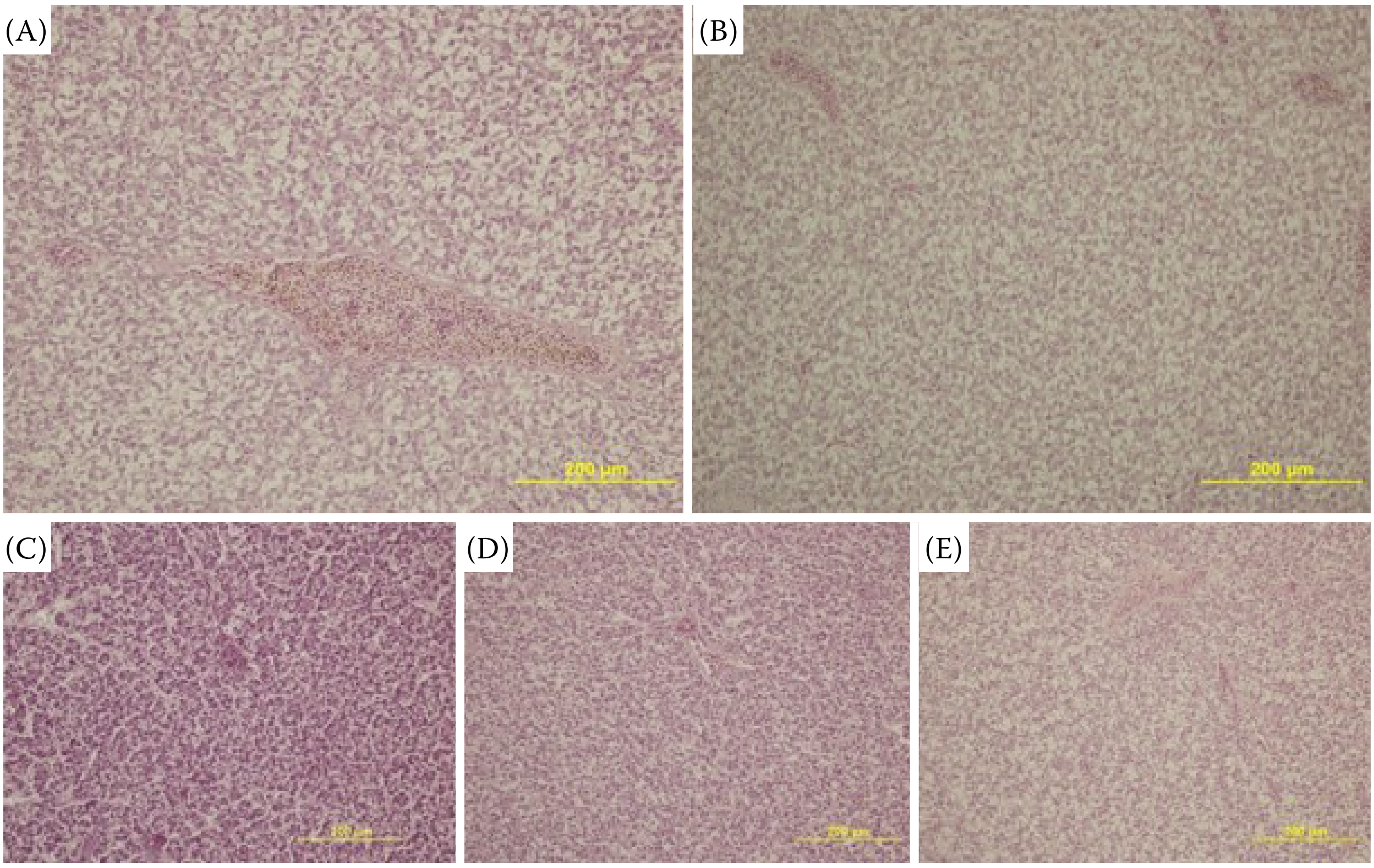

Figure S4. Representative histological pictures of the liver of *O. mykiss* after six weeks of oral exposure to PS particles ($PS\text{-}HBCD_{free}$), HBCD ($HBCD_{only}$) or their combination – $PS\text{-}HBCD_{low}$ (i.e. HBCD in c = 0.2 mg/g) and $PS\text{-}HBCD_{high}$ (i.e. HBCD in c = 1.0 mg/g)

Control group without pathological findings except for mild congestion (A); mild congestion and widespread dystrophy in $PS\text{-}HBCD_{free}$ group (B); mild congestion and focal dystrophy in $PS\text{-}HBCD_{low}$ group (C); mild congestion and widespread dystrophy in $PS\text{-}HBCD_{high}$ group (D); mild congestion and widespread dystrophy in $HBCD_{only}$ group (E). Magnification 100–200×, *n* = 6/group

https://doi.org/10.17221/95/2025-VETMED

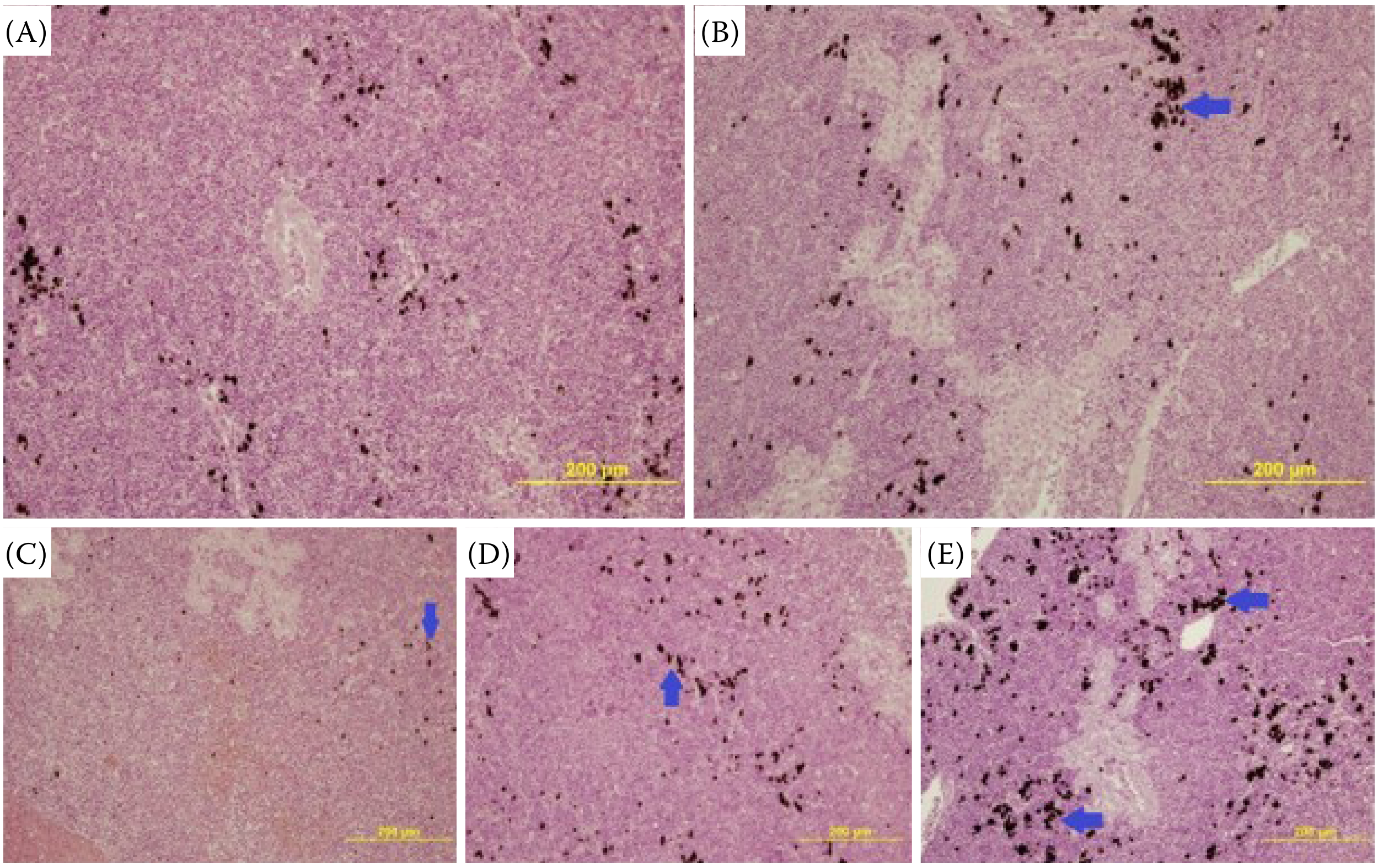

Figure S5. Representative histological pictures of the cranial kidney of *O. mykiss* after six weeks of oral exposure to PS particles (PS-$HBCD_{free}$), HBCD ($HBCD_{only}$) or their combination – PS-$HBCD_{low}$ (i.e. HBCD in c = 0.2 mg/g) and PS-$HBCD_{high}$ (i.e. HBCD in c = 1.0 mg/g)

Mild tissue congestion and local pigment deposits in control (A); PS-$HBCD_{free}$ (B); and PS-$HBCD_{low}$ (C); higher presence of pigment deposits in PS-$HBCD_{high}$ (D); and especially $HBCD_{only}$ group (E). Magnification 100–200×, *n* = 6/group

REFERENCES

Foltz JR, Plant KP, Overturf K, Clemens K, Powell MS. Detection of Nucleospora salmonis in steelhead trout, Oncorhynchus mykiss (Walbaum), using quantitative polymerase chain reaction (qPCR). J Fish Dis. 2009; 32(6):551-5.

Giraudo M, Douville M, Letcher RJ, Houde M. Effects of food-borne exposure of juvenile rainbow trout (Oncorhynchus mykiss) to emerging brominated flame retardants 1,2-bis(2,4,6-tribromophenoxy) ethane and 2-ethylhexyl-2,3,4,5-tetrabromobenzoate. Aquat Toxicol. 2017 May;186:40-9.

Hodkovicova N, Hollerova A, Caloudova H, Blahova J, Franc A, Garajova M, Lenz J, Tichy F, Faldyna M, Kulich P, Mares J, Machat R, Enevova V, Svobodova Z. Do food-borne polyethylene microparticles affect the health of rainbow trout (Oncorhynchus mykiss)? Sci Total Environ. 2021 Nov 1;793:148490.

Meiler KA, Cleveland B, Radler L, Kumar V. Oxidative stress-related gene expression in diploid and triploid rainbow trout (Oncorhynchus mykiss) fed diets with organic and inorganic zinc. Aquaculture. 2021 Feb 25; 533:736149.

Quesada-Garcia A, Valdehita A, Kropf C, Casanova-Nakayama A, Segner H, Navas JM. Thyroid signaling in immune organs and cells of the teleost fish rainbow trout (Oncorhynchus mykiss). Fish Shellfish Immunol. 2014 Jul;38(1):166-174.

Skillman AD, Nagler JJ, Hook SE, Small JA, Schultz IR. Dynamics of 17alpha-ethynylestradiol exposure in rainbow trout (Oncorhynchus mykiss): absorption, tissue distribution, and hepatic gene expression pattern. Environ Toxicol Chem. 2006 Nov;25(11):2997-3005.
